# Do Primary Emotions Predict Psychopathological Symptoms? A Multigroup Path Analysis

**DOI:** 10.3389/fpsyt.2019.00610

**Published:** 2019-08-30

**Authors:** Jürgen Fuchshuber, Michaela Hiebler-Ragger, Adelheid Kresse, Hans-Peter Kapfhammer, Human Friedrich Unterrainer

**Affiliations:** ^1^Center for Integrative Addiction Research (CIAR), Grüner Kreis Society, Vienna, Austria; ^2^University Clinic for Psychiatry and Psychotherapeutic Medicine, Medical University of Graz, Graz, Austria; ^3^Institute for Pathophysiology und Immunology, Medical University of Graz, Graz, Austria; ^4^Department of Religious Studies, University of Vienna, Vienna, Austria

**Keywords:** primary emotions, path analysis, depression, substance use disorder, anxiety disorder, somatization

## Abstract

**Background:** Research involving animal models has repeatedly proposed dysregulations in subcortically rooted affective systems as a crucial etiological factor in the development of a variety of psychiatric disorders. However, empirical studies with human participants testing these hypotheses have been sparse. Associations between primary emotions systems and different psychiatric symptoms were investigated in order to gain insights into the influence of evolutionary-rooted primary emotions on psychopathology.

**Material and Methods:** The community sample included 616 adults (61.9% female). 243 reported a psychiatric lifetime diagnosis. By applying path analysis, we estimated paths between SEEKING, ANGER, FEAR, SADNESS, CARE, and PLAY (Affective Neuroscience Personality Scales; ANPS) and symptoms of substance abuse (Alcohol, Smoking, and Substance Involvement Screening Test; ASSIST) as well as depression, anxiety, and somatization (Brief Symptom Inventory; BSI-18). To examine the moderator effects of gender and psychiatric lifetime diagnosis, multigroup analysis was applied.

**Results:** Substance abuse was associated with male sex (β = −.25), SADNESS (β = .25), and ANGER (β = .10). Depression was associated with SADNESS (β = .53), FEAR (β = .10), SEEKING (β = −.10), and PLAY (β = −.15). Anxiety was linked to SADNESS (β = .33), FEAR (β = .21) and PLAY (β = −.10). Somatization was associated with SADNESS (β = .26) and PLAY (β = −.12; all *p* < .001). Multigroup analysis revealed no differences in paths between tested groups (all *p* > .01). The model explained 14% of the variance of substance abuse, 52% of depression, 32% of anxiety, and 14% of somatization.

**Conclusions:** The results further our understanding of the differential role of primary emotions in the development of psychopathology. In this, the general assumption that primary emotion functioning might be a valuable target in mental health care is underlined.

## Introduction

Substance use disorder (SUD) is generally defined as a chronic and pathological and compelling urge to consume one or more psychoactive substances despite harmful effects for oneself and others (1). According to the World Drug Report 2017, problematic substance use and SUDs currently affect about 29.5 million people (2). Hence, they pose a serious threat not only to individual health but also significantly burden public health systems. Furthermore, SUDs show substantial comorbidities with a wide range of psychiatric disorders (3). An exceptionally prevalent relationship seems to exist with regards to mood disorders like depression and anxiety disorders (4). Moreover, despite considerable overlap between withdrawal symptoms related to SUDs and somatoform disorders, few studies have investigated the comorbidity between SUDs and somatization (5). However, several studies report a substantial association between both disorders (5, 6).

Predominantly based on animal models, affective neuroscience (AN) theory proposes dysregulations in subcortical affective systems as an important factor in the etiology of a variety of psychiatric disorders (7, 8). Currently AN and neuropsychoanalytic researchers distinguish seven primary emotion networks which arise from the periaqueductal gray and expand into the limbic forebrain (8, 9). Four of those systems have evolved from reptilian roots (10). These phylogenetically oldest networks consist of the SEEKING, FEAR, LUST, and ANGER systems. Moreover, three primary emotion networks specifically manifest in evolutionarily higher species like mammalians and certain birds (10). These networks include the PANIC/GRIEF or SADNESS, CARE, and PLAY systems (8, 10, 11).

With regard to personality psychology, the Affective Neuroscience Personality Scales (ANPS) (12) have been developed to measure individual dispositions toward Panksepp, (11) primary emotions circuits. The ANPS assesses six facets of primary emotion dispositions, including SEEKING, CARE, PLAY, FEAR, SADNESS, and ANGER but does not measure LUST due to conceptual concerns (12, 13). In line with Panksepp (7), it might be argued that individual differences in primary emotion dispositions are able to explain clinically significant aspects in the development of psychiatric disorders.

Largely in consensus with Berridge (14, 15), AN theory proposes that SUD is characterized by pathological changes within the SEEKING/mesolimbic-dopamine system. In the course of this disorder, the SEEKING network is increasingly and, ultimately, predominantly activated in association with substance-related appetitive memories, substance consumption, and the desire to alleviate negative affective states (16–18). Furthermore, there is strong evidence that certain individuals may be predisposed to addiction through certain psychological and neural parameters, such as hyperexcitability of the brain stress system or depressiveness. In turn, this might promote the reorganization of SEEKING toward drugs or other addictive behaviors like gambling (16).

In addition, it is assumed that SUDs are associated with perturbations within the LUST and PANIC/GRIEF network (18, 19). In correspondence to this, dopamine surges of the artificially excited SEEKING system might not be the primary object of addiction, but rather the feeling of reward itself, mediated in large part by the predominantly opioid controlled LUST and PANIC/GRIEF systems. Furthermore, the neurobiology of attachment in mammalians, primarily mediated by the PANIC/GRIEF system, and SUDs share striking similarities which are mirrored by a significant overlap in behavioral aspects of both social dependence and addiction (11, 18, 20–22). Common neurochemical sites of action and change regarding attachment and addiction development include dopamine D1 and D2 receptors; mu-, delta-, and kappa-opioid receptors; and corticotropin-releasing factor (20).

Behavioral similarities between attachment/loss and addiction/withdrawal include: social bonding/drug dependence, drug tolerance/estrangement, and drug withdrawal/separation distress (11). Therefore, addiction is often conceptualized as a deranged form of attachment (18, 19). Furthermore, the behavioral aspects of opioid withdrawal show especially strong resemblances to separation distress, comprising psychological and somatic pain, crying, loss of appetite, depression, insomnia, and aggressiveness (11). In this context, addiction might be understood as a dysfunctional attempt to compensate for overwhelming feelings of isolation, loss, and sadness mediated by an overactive PANIC/GRIEF system.

Until now the role of other primary emotion systems in the emergence of addiction cycles has been largely neglected in AN theory and research. However, Unterrainer et al. (23) were able to show increased SADNESS, FEAR, and ANGER in patients suffering from polydrug use disorder compared to healthy controls. Moreover, very little is known about the role of PLAY and CARE in addiction etiology. With regard to the neurochemistry of PLAY, which relies on the endogenous cannabinoid system (8), it might be plausible to assume that PLAY is involved in cannabis addiction. However, this assumption lacks empirical support. Similarly, so far, there is no data suggesting the significance of CARE in SUD development in humans (23). Nevertheless, animal research showed that lactating dams exhibited reduced brain activity in the mesolimbic-dopamine system—compared to virgin females—if the animals were exposed to cocaine (24). In general, it is still unclear if addiction might be a self-medication strategy against negative affects in general, as suggested by other authors [e.g., Ref. (25)], rather than a more specific coping mechanism against increased PANIC/GRIEF and decreased SEEKING as proposed in AN theory (18, 19). In this context, SUD patients might use drugs as an artificial defense mechanism against overwhelming, often undifferentiated, perceived affects in general. Hence, the tendency toward depression and anxiety—frequently observed in SUD patients—is often somatized, unverbalized, and experienced as physical pain (26, 27).

In correspondence to this, AN theory conceptualizes depression as an evolutionarily conserved mechanism in which the overactive PANIC/GRIEF system shuts down the acute panic or protest phase of separation distress and triggers a state of *despair* which is characterized by sustained overactive GRIEF and discontinuation of the SEEKING system, experienced as intense dysphoria (28). Furthermore, a study by Montag et al. (29), applying the ANPS, suggested associations between depression and increased dispositions to SADNESS and FEAR, as well as decreased SEEKING and PLAY. With regard to anxiety disorders, Panksepp (7, 30) proposes a hyperactivation of the FEAR system, which is related to either pathologically increased activation of the amygdala or a corresponding deactivation of the prefrontal cortex (31). Moreover, Panksepp (11) suggests a clinically significant relationship between the emergence of anxiety disorders and hypoactivity of the PANIC/GRIEF or SADNESS network. However, until now, quantitative-empirical research regarding the relationship between primary emotion dispositions and anxiety disorders has been largely neglected.

**Figure 1:**
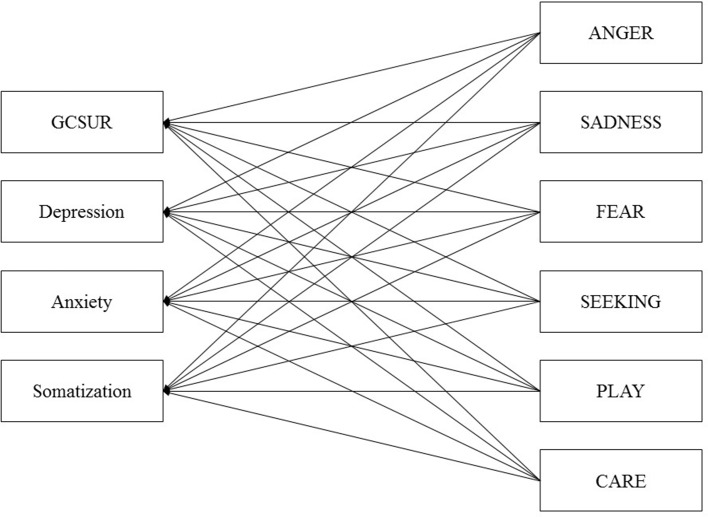
Initial model of primary emotions and psychiatric symptoms controlled for Age and Sex. GCSUR, Global Continuum of Substance Use Risk.

Similarly, to the best of our knowledge, primary emotion networks underlying somatization have not been investigated yet by studies applying standardized questionnaires. From a psychodynamic point of view, somatization is understood as a defense against otherwise unbearable affects (32, 33). With regard to the shared neuronal architecture of pain processing and social isolation, somatization has been linked to increased activity of the SADNESS system (32, 34). To further investigate the clinical significance of AN framework, the present study applied path analysis to examine the relationship between psychopathological symptoms (SUD, depression, anxiety disorder, and somatization) and different dimensions of primary emotions (SEEKING, FEAR, ANGER, SADNESS, PLAY, and CARE). The conceptual framework is outlined in **Figure 1**. Furthermore, by applying multigroup path analysis, this study tested possible moderator effects of gender and psychiatric lifetime diagnosis.

## Material and Methods

### Procedure

Participants were recruited through advertising on social networks, including public forums and announcements at the University of Graz, Austria. After declaring informed consent, each participant was asked to fill out a range of demographic questions (e.g., age, sex, education status, and lifetime psychiatric diagnosis) as well as a variety of standardized questionnaires, including the Affective Neuroscience Personality Scales, the Brief Symptom Inventory, and the Alcohol, Smoking, and Substance Involvement Screening Test. The data was acquired *via* the online-survey platform LimeSurvey^©^. Participants were included if they spoke German fluently, filled in all questionnaires and were aged between 18 and 69 years. In correspondence to this, 874 discontinued the participation before completion while 12 participants did not meet the required age for participation. The study was carried out in accordance with the Declaration of Helsinki. Ethical approval was granted by the Ethics Committee of the Medical University of Graz, Austria. The recruitment of participants was carried out between April 2017 and March 2018.

## Psychometric Assessment

### Primary Emotions

*The Affective Neuroscience Personality Scales* (ANPS) (12) [German version by Reuter and Hennig (35)] [see Ref. (36) for a more recent version] is a self-report measurement which operationalizes behavioral traits related to the concept of subcortical primary emotion circuits, developed by Panksepp (11). The questionnaire includes the following subscales: SEEKING, SADNESS, FEAR, RAGE, CARE, and PLAY. The additional scale for “spirituality” was not analyzed in the course of this study. The ANPS is comprised of, overall, 110 items with 14 items for each subscale and is rated on a four-point Likert scale ranging from 1 (“strongly disagree”) to 4 (“strongly agree”). SEEKING summarizes the disposition toward feelings of positive curiosity toward new experiences, the tendency to explore, and a sense of being able to achieve relevant goals. ANGER is conceptualized by the trait of being easily frustrated and irritated, the frequent expression of anger in a verbal or physical way, the experience of being angry due to frustrations, and being unable to calm down. FEAR measures the individuals’ tendency toward feelings of anxiety, tenseness, worries, and ruminations. SADNESS operationalizes the tendency of feeling separation distress, loneliness, and sorrow. CARE operationalizes the individual’s tendency toward feelings of empathy, caring for children, people in need and animals, and a general enjoyment of being needed by others. PLAY measures the trait of being protracted toward games with physical contact, laughter, fun, as well as being generally happy and joyful. All scales showed acceptable to good internal consistencies, with Cronbach’s alpha ranging from 0.78 (SADNESS) to 0.89 (SEEKING).

### Psychiatric Symptoms

The *Alcohol, Smoking, and Substance Involvement Screening Test* (ASSIST) (37) is a standardized interview which is used to assess psychoactive substance use and related problems. This questionnaire measures lifetime use and substance-related symptoms of 10 substance groups including tobacco, alcohol, cannabis, cocaine, amphetamines, inhalants, sedatives, hallucinogens, opioids, and “other drugs.” Questions 2–5 are rated on a seven-point Likert scale ranging from 0 (“never”) to 6 (“daily or almost daily”). These scales assess the “frequency of drug use,” “craving to use the drug,” “problems” (health, social, legal, or financial) because of drug use, and “failed expectations.” Moreover, questions 6, 7, and 8 are rated on a three-point scale (0 = “no, never”; 3 = “yes, but not in the past 3 months”; 6 = “yes, in the past 3 months”) and cover “expressed concerns by relatives or friends,” “failed attempts to cut down drug use,” and “drug injection.” For this study, the total score “Global Continuum of Substance Use Risk” (GCSUR) was calculated. This scale showed an acceptable internal consistency with Cronbach’s alpha = 0.78.

The *Brief Symptom Inventory* (BSI-18) (38) [German version: Ref. (39)]. The BSI-18 consists 18 items assessing the amount of symptom burden over the past 7 days. The BSI-18 includes the subscales depression, anxiety, and somatization. Items are rated on a five-point Likert scale ranging from 0 “absolutely not” to 4 “very strong.” A total score “Global Severity Index” can be generated by adding the scores of every item. All scales showed good internal consistencies, with Cronbach’s alpha ranging from 0.80 (somatization) to 0.91 (depression).

### Statistical Analysis and Analysis Strategy

The path analysis estimations and multigroup path analysis were conducted *via* AMOS 18. SPSS 21.0 was used for data management and descriptive statistics. Initially, bivariate correlations were calculated to assess the strength of relations among all variables. In a next step data was fitted to an initial path model that included the following paths: all primary emotions to GCSUR, depression, anxiety, and somatization (**Figure 1**). This model was controlled for age and sex. Furthermore, correlations between the disturbance terms amongst individual primary emotions and psychiatric symptoms were assigned. After the initial model was fitted, a pruning strategy was applied by removing non-significant paths from primary emotions to psychiatric symptoms. The path models were estimated using the maximum likelihood method in AMOS.

In accordance with Kline (40), the following fit indices were considered as markers for an acceptable model fit: (a) the comparative fit index (*CFI*) > 0.90, (b) Tucker-Lewis index (*TLI*) relative fit index > 0.90, (c) the square root error of approximation (*RMSEA*) < 0.08, and the upper bound of its 90% confidence interval < 0.1. For the comparison of competing models, the Akaike’s information criterion (*AIC*) was used, with the smaller value indicating better fit. The alpha-level was set to 0.01. To test for possible moderator effects of sex and self-reported psychiatric lifetime diagnosis, multigroup analysis was performed (41). In order to statistically evaluate the differences in path coefficients across the groups, tests of invariance with a chi-square difference test were performed. A chi-square corresponding to a probability level of less than.01 was the criterion by which the null hypothesis that the relevant parameters were equal across the groups (female *vs*. male; participants without a lifetime psychiatric diagnosis *vs*. participants reporting a lifetime psychiatric diagnosis) was rejected.

## Results

### Sample Characteristics and Descriptive Statistics

The investigated community sample was comprised of 616 German-speaking adults (381 female, 61.9%), ranging in age from 18 to 69 years (*M* = 30; *SD* = 9.53). In this study, 231 (37.5%) participants declared a university degree as their highest educational level. Two hundred fourteen (34.7%) stated a general qualification for university entrance, 46 (7.4%) a high school degree, and 96 (15.5%) participants stated a completed apprenticeship as their highest educational level. Twenty-nine (4.7%) participants stated that they left school without graduation. Regarding the current occupation of participants, 222 (36%) were in employment, 313 (50.8%) in education, 57 (9.2%) were unemployed, and 24 (3.8%) were on pensions. Concerning the current relationship status, 59 (9.6%) were married, 259 (42.0%) in a relationship, and 298 (48.4%) were single. The nationality of most participants was either German (*n* = 334; 54.5%), Austrian (*n* = 218; 35.5%), or Swiss (*n* = 30; 4.8%), while 34 (5.5%) had other nationalities. Finally, 243 (39.4%) participants declared that they had been diagnosed with a (lifetime) psychiatric disorder. The majority of these participants were diagnosed with depression (*n* = 147; 60%) and 50 (21%) with other affective disorders, and 46 (19%) participants were diagnosed with other psychiatric disorders. As shown in **Table 1**, participants with and without a psychiatric diagnosis differed (*p* < 0.001; η^2^ = 0.03–0.15) in every examined variable with the exception of CARE (*p* = n.s.).

**Table 1:** Descriptive statistics, sex differences, and differences between healthy and diagnosed participants and interaction effects among examined variables.

		Female		Male		Healthy		Diagnosis		Sex	Health	Gender x health
Measure	α	*M*	*SD*	*M*	*SD*	*M*	*SD*	M	SD	F(3, 612)	F(3, 612)	F(3, 612)
BSI-18												
Depression	0.91	13.55	6.77	13.39	6.67	11.39	5.61	16.71	7.03	0.18	96.67**	0.27
Anxiety	0.81	12.24	5.12	11.37	4.51	10.81	4.24	13.60	5.37	3.08	42.42**	0.67
Somatization	0.80	10.70	4.41	9.88	4.07	9.63	4.04	11.56	4.43	3.22	27.14**	0.02
ASSIST												
GCSUR	0.78	34.26	32.31	48.99	37.82	33.56	34.51	49.57	34.13	31.40**	36.17**	0.02
ANPS												
SEEKING	0.75	2.82	0.42	2.80	0.37	2.89	0.38	2.70	0.42	2.13	35.91**	1.51
FEAR	0.89	2.86	0.55	2.71	0.55	2.64	0.52	3.06	0.51	7.54*	86.21**	0.06
ANGER	0.85	2.62	0.52	2.56	0.49	2.53	0.48	2.71	0.53	1.44	15.36**	0.01
SADNESS	0.78	2.72	0.45	2.57	0.45	2.52	0.41	2.87	0.43	12.72**	79.87**	2.46
PLAY	0.76	2.79	0.49	2.76	0.47	2.89	0.45	2.60	0.48	1.71	53.82**	0.04
CARE	0.83	2.99	0.42	2.69	0.40	2.90	0.41	2.85	0.47	81.87**	5.62	1.67

n = 616; * p < .01; **p < .001; BSI-18, Brief Symptom Inventory; ASSIST, The Alcohol, Smoking and Substance Involvement Screening Test; ANPS, Affective Neuroscience Questionnaire Scales.

As shown in **Table 2**, all negative primary emotion dispositions (SADNESS, FEAR, and ANGER) showed positive correlations with every assessed psychiatric variable (GCSUR, depressive symptoms, anxiety symptoms, and somatization) (all *p* < 0.001), whereas CARE did not correlate with any clinical marker (all *p* > 0.01). Moreover, PLAY and SEEKING, which showed substantial intercorrelations (r = .56; *p* < 0.001), were negatively correlated with depressive symptoms, anxiety symptoms, and somatization (*p* < 0.001); however, neither were correlated with GCSUR (*p* > 0.01). Finally, male sex was positively correlated with GCSUR (*r* = .20; p < 0.001), while sex had no significant relationship to other investigated psychiatric symptoms (p > 0.01).

**Table 2:** Descriptive statistics and correlations among examined variables.

Variable	1	2	3	4	6	7	8	9	10	11	12
1. Global continuum of substance risk	–										
2. Depression	.44*	–									
3. Anxiety	.40*	.69*	–								
4. Somatization	.38*	.52*	.67*	–							
5. SEEK	−.12	−.37*	−.19*	−.15*	–						
6. FEAR	.19*	.59*	.53*	.33*	−.33*	–					
7. ANGER	.19*	.25*	.27*	.24*	−.09	.34*	–				
8. SADNESS	.26*	.69*	.53*	.35*	−.32*	.73*	.37	–			
9. CARE	−.08	−.08	.01	.01	.28*	.09	−.06	.06	–		
10. PLAY	−.10	−.45*	−.29*	−.22*	.56*	−.39*	−.11	−.41*	.41*	–	
11. Sex	−.20*	−.01	.08	.00	.03	.14*	.06	.15*	.34*	.03	–
M or N	39.89	13.49	11.91	10.39	2.81	2.81	2.60	2.66	2.88	2.78	381
SD or %	35.22	6.72	4.91	4.30	0.40	0.55	0.51	0.45	0.43	0.48	61.9

n = 616; * p < .001; Sex was coded as: 0 = male; 1 = female.

### Path Analysis

**Figure 2:**
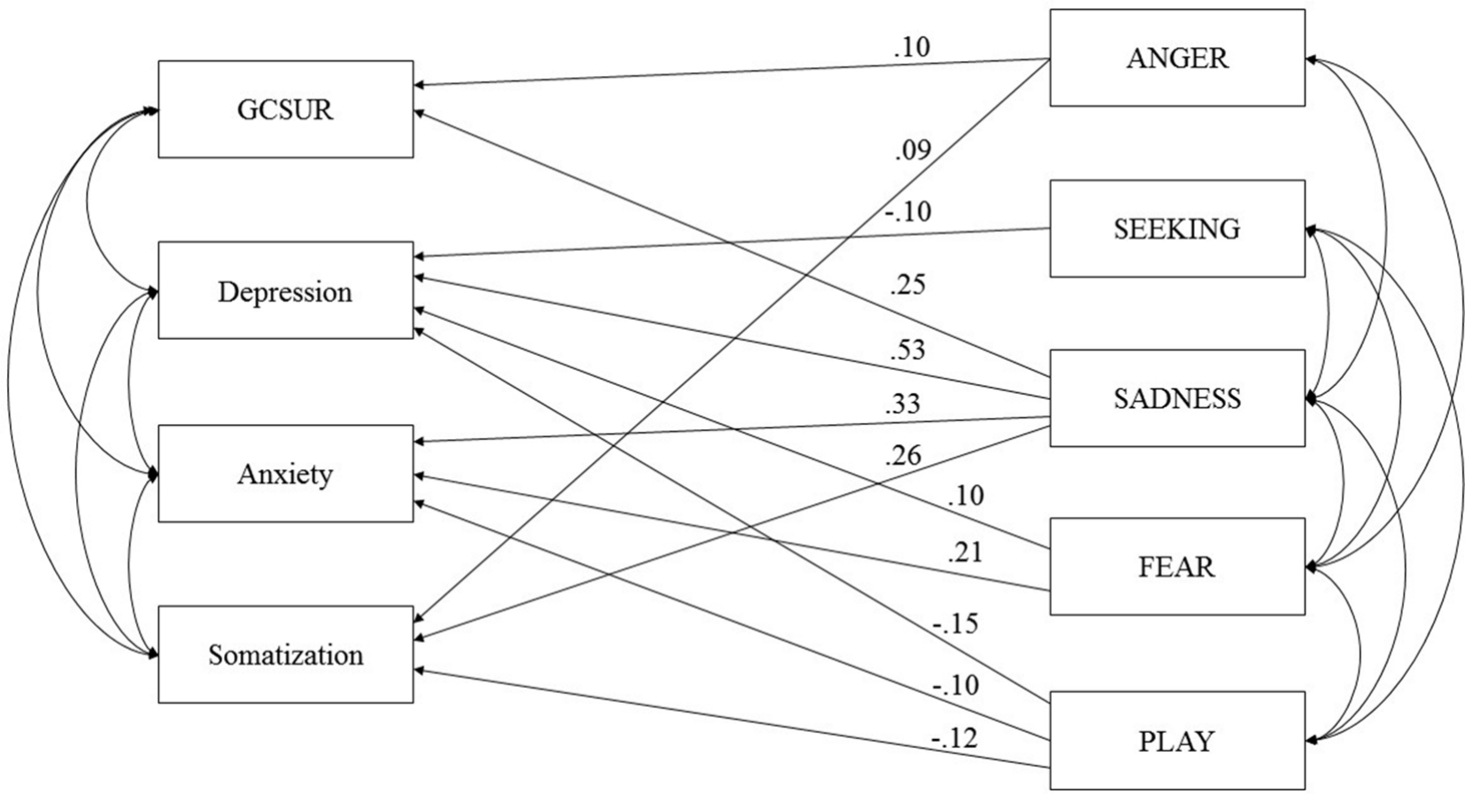
Final model of primary emotions and psychiatric symptoms controlled for Age and Sex. GCSUR, Global Continuum of Substance Use Risk; *p < 0.001; curved arrows indicate significant correlations (p < 0.001).

The initially proposed model (see **Figure 1**), which was controlled for sex and age, showed a poor fit due to insufficient RMSEA values: *RMSEA* = 0.07 (90% CI: 0.03, 0.12), *TLI* = 0.92, *CFI* = 1.00, and *AIC* = 186.60. Therefore, a second model was tested which excluded CARE, as this dimension of primary emotions did not correlate with the clinical variables. The second model showed a poor fit as well: *RMSEA* = 0.09 (90% CI: 0.04, 0.14), *TLI* = 0.90, *CFI* = 1.00, and *AIC* = 160.95. As a third step, the second model was trimmed by deleting all non-significant paths between variables (see **Figure 2**). This included: (a) paths between ANGER, depressive symptoms, and anxiety symptoms; (b) paths between SEEKING, GCSUR, anxiety symptoms, and somatization; (c) paths between FEAR, global continuum of substance risk and somatization; and (d) paths between PLAY and global continuum of substance risk.

The third model showed an acceptable fit: RMSEA = 0.05 (90% CI: 0.03, 0.08), TLI = 0.97, and CFI = 0.99 *AIC* = 159.74. The trimmed model suggested the following associations: GCSUR was associated with male sex (β = −.25), SADNESS (β = .25), and ANGER (β = .10); depressive symptoms were associated with increased SADNESS (β = .53) and FEAR (β = .10) and decreased dispositions to SEEKING (β = −.10) and PLAY (β = −.15); anxiety symptoms were related to increased SADNESS (β = .33), FEAR (β = .21), and decreased PLAY (β = −.10); and somatization was linked to increased SADNESS (β = .26) and ANGER (β = .09) and decreased PLAY (β = −.12) (all *p* < .01).

In summary, the final model was able to explain 14% of the variance of global substance risk, 52% of depressive symptoms, 32% of anxiety symptoms, and 14% of somatization.

Furthermore, to examine the possible moderation effects of psychiatric lifetime diagnosis and sex, additional multigroup analysis was conducted. The comparison between groups—which were conducted *via* chi-square difference tests (female *vs*. male; participants without a lifetime psychiatric diagnosis *vs*. participants reporting a lifetime psychiatric diagnosis)—revealed no statistically significant difference between paths (χ² = 0–4.788; all *p* > 0.01). The unconstrained multigroup analysis model exhibited the following fit indices: *RMSEA* = 0.02 (90% CI: 0.01, 0.03), *TLI* = 0.98, and *CFI* 0.99; *AIC* = 505.94.

## Discussion

This study investigated the relationship between symptoms of psychiatric disorders and primary emotions. In contrast to Fuchshuber et al. (42), which followed a confirmatory approach focusing on the role of the primary emotion despair in SUDs and depressive symptoms (18), the present study applied path analysis to investigate the relationship between all primary emotion dimensions, SUD, and other psychiatric disorders in a more exploratory manner.

Our results suggest that SUD symptoms are associated with increased SADNESS and, to a lesser extent, with increased ANGER. These findings echo previous results by Unterrainer et al. (23) which indicated increased SADNESS, FEAR, and ANGER dispositions in SUD inpatients. However, with regard to the relatively small percentage of overall explained variance, SUD might be less related to primary emotions than previously expected. This is particularly the case for SEEKING, which, in line with Unterrainer et al. (23), did not show significant associations with SUD symptoms. This finding, which contradicts evidence from neuroscientific research (16, 17, 43), might be explained by conceptual differences between functional aspects of the ML-DA or SEEKING system and the general disposition toward SEEKING measured by the ANPS. More specifically, with regards to its role in reinforcement learning, the ML-DA/SEEKING network seems crucial in the development of SUD. However, this might not be reflected in the individual’s disposition toward decreased SEEKING. Furthermore, our results were gathered in the course of a cross-sectional study; hence, it is impossible to infer causal conclusions based on our results. Therefore, it is conceivable that many forms of SUD can be understood as dysfunctional coping strategies against a hypoactive SEEKING system as outlined by Zellner et al. (18) and Solms et al. (19). Yet, owing to the cross-sectional study design, we might have been unable to detect this association, as problematic consumption of psychoactive substances might have artificially increased the individual’s SEEKING disposition (8). Thus, in order to sufficiently investigate the relationship between SEEKING and SUD, it will be necessary to conduct longitudinal studies assessing SEEKING prior to the onset of problematic substance use.

In contrast, our findings highlight the role of SADNESS and ANGER in SUD. In line with the results of Unterrainer et al. (23), this partly supports assumptions of AN theory (18, 19) and reaffirms observations of object relations theory emphasizing the etiological role of aggression in SUD (44, 45). This finding further supports the notion of substance abuse as a function of artificial affect regulation (26). By taking drugs, the addicted individual tries to seal gaps in a corrosive personality structure (42), which is linked to increased negative affects (46, 47). Specifically, addictive behaviors seem to be associated with increased feelings of loneliness and isolation but also with heightened feelings of rage and aggression, which both are experienced by the SUD patient as intensely unpleasurable and ultimately overwhelming (8, 11).

The observed relationship between SUD and SADNESS further highlights the conceptualization of addiction as an attachment disorder, specifically linked to dysregulations within the endogenous opioid system (20, 21, 48). Furthermore, the association between SUD and ANGER may support psychoanalytic theories that relate substance abuse to auto-aggressive behavior, which is presumably directed against malicious inner self and object representations (44, 45, 49).

Moreover, our findings suggest a differential role of primary emotions in the development of psychopathology. Thereby, SADNESS seems to play a substantial role in all investigated disorders. However, in contrast to SUD, depressive symptoms were also predicted by decreased PLAY and SEEKING and increased FEAR, which is largely in line with findings from Montag et al. (29). These findings highlight the basic assumption of AN depression theory regarding the central role of a negative cascade between hyperactive SADNESS and hypoactive SEEKING system in depression etiology, as well as amplify the affective and neurophysiological complexity of depression (28, 50).

In addition, a similar pattern was found for anxiety symptoms, which were associated with increased SADNESS, FEAR, and decreased PLAY. In correspondence to this, the observed association between SADNESS and symptoms of anxiety disorders reflect results of a recent meta-analysis by Kossowsky et al. (51), which concluded that separation anxiety disorder in childhood significantly increases the risk of anxiety disorders in adulthood. With regard to Panksepp (9), conceptualization of the neuroarchitecture of the SADNESS system, the link between SADNESS, and anxiety disorders might be based on similar neurological correlates, including the amygdala and the anterior cingulate cortex (52, 53). Additionally, our results not only support Panksepp (7) hypothesis regarding the importance of the PANIC/GRIEF or SADNESS system in anxiety disorders, but also highlight his emphasis on the clinical significance of PLAY, which has been traditionally neglected in psychiatric research (8, 9).

Likewise, this assumption is reaffirmed in the observed association between PLAY and somatization symptoms. Taken together, these findings might be linked to the predominance of negative primary emotions, which inhibit the functional activity of the PLAY circuit (8, 29). Furthermore, the significant relationship between increased SADNESS and somatization might reflect the relationship between SADNESS and the endogenous opioid system, as a hypoactivity of mu and delta opioid network—correlated to increased SADNESS—is known to promote feelings of bodily discomfort (9, 20). Nevertheless, self-rated primary emotion dispositions explained only a small fraction of the somatization symptom variance. This finding resonates with several studies indicating that somatization patients showed increased alexithymia scores (54–56).

In addition, the results of the multigroup analysis indicated no significant sex differences as well as no differences between healthy and diagnosed participants regarding the strength and direction of the relationship between symptoms and primary emotion functioning. These results support and expand findings by Montag et al. (29), which suggested a continuum model regarding the relationship between primary emotions and depression in healthy participants as well as clinically treated patients. Moreover, our findings suggest that there are no sex specific differences between the associations of the ANPS and symptoms of SUD, depression, anxiety, and somatization.

## Limitations

The present study reanalyzed an extended sample partially already investigated in Fuchshuber et al. (42). Therefore, the results of our analysis should not be interpreted as independent evidence. Moreover, a question asked the participants to report if they have ever been diagnosed with a psychiatric disorder by a licensed psychiatrist and a follow-up question assessed the specific diagnosis, which limits the descriptive value of our data regarding psychiatric diagnoses. Therefore, future research should aim at assessing psychiatric diagnoses in more detail by applying standardized interviews.

Furthermore, as there is no validated measure for the assessment of LUST currently available, it is impossible to estimate the clinical relevance of this primary emotion system. Despite having a key role in AN- and neuropsychoanalytic theory, LUST was not included in the ANPS, as its authors claimed that people would not be open enough to report about their sexuality (12). However, this assumption seems questionable, especially with regards to the variety of self-report measures of sexuality already existing. Hence, future research should aim at developing a self-report measure for LUST, to fully map the AN framework and its role in psychiatric etiology.

In addition, the present study assessed substance-related problems by means of the global continuum of substance use risk (37). However, problematic consumption of different substance classes might be associated with differential primary emotion dysregulations (18). Hence, future studies should investigate the affective profiles for specific substance-related problems. Moreover, it should be kept in mind that our results suggest substantial intercorrelations between SUD and symptoms of mood disorders, as well as between different dimensions of primary emotions. Therefore, the interplay between other psychiatric symptoms and primary emotions underlying SUD should be understood as a complex and interdependent phenomenon. Finally, due to the cross-sectional design of this study, the results of the path analytic models presented herein are associative in nature and do not allow for causal interpretations.

## Conclusion

The present study was able to gather empirical evidence for the psychiatric significance of primary emotion dispositions. Our results indicate that specific pattern of primary emotion dispositions underlie symptoms of SUDs and other psychiatric disorders. Hence, primary emotions might serve as a valuable target in the psychotherapeutic process. In correspondence to this, our findings present a tentative roadmap for neuroscientists as well as clinical researchers, underscoring primary emotion networks which might deserve attention in future research.

## Data Availability

The raw data supporting the conclusions of this manuscript will be made available by the authors, without undue reservation, to any qualified researcher.

## Ethics Statement

This study was carried out in accordance with the recommendations of the ethics guidelines of the Medical University of Graz. The protocol was approved by the ethics committee of the Medical University of Graz. All subjects gave written informed consent in accordance with the Declaration of Helsinki.

## Author Contributions

JF and HU conceptualized the study. JF conducted data collection and data evaluation. JF wrote the manuscript draft. HU, AK, MH-R and H-PK supervised the drafting of the manuscript. All authors proofread the final version of the manuscript and gave their consent for publication.

## Conflict of Interest Statement

The authors declare that the research was conducted in the absence of any commercial or financial relationships that could be construed as a potential conflict of interest.
